# A New Resistant Starch Material Obtained from Faba Beans (*Vicia faba* L. Creole): Potential Modulation of the Diabetic Condition in Diabetic Wistar Rat Model

**DOI:** 10.3390/nu17233807

**Published:** 2025-12-04

**Authors:** Teodoro Suárez-Diéguez, Mariza Olvera Nájera, Mariana Silva, Guadalupe López-Rodríguez, José Alberto Ariza-Ortega, Aurora García-Tejedor, Juan Antonio Nieto

**Affiliations:** 1 Área Académica de Nutrición, Instituto de Ciencias de la Salud, Universidad Autónoma del Estado de Hidalgo, Abasolo 600, Colonia Centro, Pachuca de Soto CP 42000, Mexico; glopez@uaeh.edu.mx (G.L.-R.); jose_ariza@uaeh.edu.mx (J.A.A.-O.); 2 Department of Biochemistry, National School of Biological Sciences, National Polytechnic Institute, Av. Luis Enrique Erro S/N, Unidad Profesional Adolfo López Mateos, Zacatenco, Alcaldía Gustavo A. Madero, México City CP 07738, Mexico; marizaolveranajera@gmail.com; 3 Bioactivity and Nutritional Immunology Group (BIOINUT), Faculty of Health Science, Universidad Internacional de Valencia (VIU), Calle Pintor Sorolla 21, E46002 Valencia, Spain; msilval@universidadviu.com (M.S.); agarciate@universidadviu.com (A.G.-T.)

**Keywords:** faba, beans, starch, resistant starch, diabetes, digestibility, hyperglycemia, oxidative stress

## Abstract

Background: Resistant starch, and specially retrograded starches (RS), have been suggested as useful biological molecules to improve the glucose management in diabetic conditions. However, the influence of the botanical origin in the RS biological capacities make necessary its evaluation, where RS from legumes have been paid less attention compared to other sources as cereals. Objectives: A RS product obtained from creole Faba beans (*Vicia faba* L. creole), was evaluated for the first time as a material capable of improving glucose homeostasis in diabetic conditions. Methods: The RS ingredient investigated (with a reduced digestibility of 50%) was tested in a Wistar rat model with induced diabetes, fed with a 15 or 30% replacement of RS ingredient in the diet. Diverse nutritional and biomarkers were analysed. Results: As a result of the reduced digestibility of the RS ingredient, diabetic animals fed with RS replacement (15% or 30%) showed attenuated postprandial hyperglycemia responses, reducing the hyperglycemic condition close to 29% compared to non-treated diabetic animals (24.56 ± 7.50 and 25.02 ± 3.54 vs. 34.65 ± 1.89 mmol/L, respectively). In addition, fasting serum glucose levels were significantly reduced (22%). Other biochemical parameters associated with glucose metabolism, such as glycosylated hemoglobin and AGEs levels, also improved. Furthermore, significant improvements in nutritional parameters (such as weight gain) and a lower insulin resistance index were determined. In contrast, no clear effects were observed in lipid metabolism and oxidative stress biomarkers in the treated group. Conclusions: The results of this research suggest that the retrograded starch from creole beans evaluated could be a potential functional food ingredient capable of enhancing glucose homeostasis in diabetic conditions.

## 1. Introduction

Diabetes mellitus is defined as a metabolic disease where hyperglycemia occurs frequently. This pathological condition happens because of reduced insulin activity due to the reduction or defects in islet β-cell function [1]. Diabetes is also characterized by increased oxidative stress and, consequently, with other metabolic syndrome pathologies, such as hypercholesterolemia, dyslipidemia, and inflammation, among others [2]. Hence, the digestibility profile, and therefore, the postprandial glycemic response, are critical factors associated with diabetes development [3].

The main source of glucose in the human diet is starch [4]. The salivary and pancreatic α-amylase hydrolyze the starch molecules, allowing the release of a high amount of glucose molecules. These unlashed glucose molecules are subsequently absorbed in the small intestine, generating a postprandial glycemic response [5]. However, starch molecules are composed of three different fractions that condition glucose release during the digestion process and, therefore, the postprandial glycemic response. Starch digestibility is a consequence of its composition in rapidly (RDS) and slowly (SDS) digestible starch fractions, conditioning the glucose releasing kinetic, total glucose released, and glycemic response. Conversely, the fraction designed as resistant starch (RS) is a crystalline fraction that cannot be hydrolyzed by digestion enzymes, remaining in the gastrointestinal tract, and being fermented by the microbiota in the colonic [4]. However, the composition of these three fractions and the starch structure depend on the botanical source, as each plant species has a particular starch fraction composition [6]. As a consequence, starch digestibility is influenced not only by the botanical source [7], but also by the technological methods applied to starchy products [6]. Specifically, starch retrogradation is an industrial process consisting of increasing the crystallized starch fraction through gelatinization and crystallization. Starch gelatinization breaks down the starch granule structure, allowing the retrogradation step to occur during cooling and storage through a continuous non-equilibrium of recrystallization process [8]. Therefore, the retrogradation process increases the fraction of resistant starch in starchy products, allowing them to generate type III resistant starch in the molecule, also known as retrograded starch [9].

The European Food Safety Authority supports a health claim for RS, recommending a daily allowance of 20 g/day [10]. However, because of the botanical sources influence the SDS, RDS, and obtained RS amount [6,7,11], it is mandatory to analyze their digestibility and postprandial glycemic response in order to evaluate the potential benefits of RS ingredients [11]. In this context, several sources of starch have been evaluated to produce retrograded RS ingredients, such as various cereals, legumes, and roots [11,12,13,14]. Nevertheless, while cereal RS has been extensively investigated, more evidence is needed to confirm that RS ingredients obtained from legume sources can serve as potential functional modulators of glucose homeostasis [11].

This investigation explores, for the first time, the potential functional capacity of RS ingredients obtained from Mexican creole faba beans (*Vicia faba* L. creole) to modulate glucose metabolism and homeostasis. In this study, the impact of an RS ingredient obtained from creole faba beans on the postprandial glucose response, nutritional status, metabolic biomarkers, and oxidative stress was evaluated for the first time. To this purpose, the potential functional capacity of the RS ingredient was evaluated in a Wistar rat model under healthy and diabetic conditions and with induced diabetes.

## 2. Materials and Methods

### 2.1. Plant Material

Faba bean (*Vicia faba* L. creole) from the Valley of Mexico was cultivated in diverse crops in this area of Mexico in 2018. Faba beans were obtained from local farmers. Prior to the sample use, the absence of biological material and the good condition of the beans were confirmed.

### 2.2. Chemicals

Pullulanase type I (EC 3.2.1.41) from *Bacillus subtilis* was obtained from Novozymes Corp. (Bagsvaerd, Denmark); sodium acetate anhydrous, streptozotocin (STZ) S0130, sodium hydroxide, sodium citrate, trichloroacetic acid, phosphate buffer (potassium phosphate monobasic, sodium phosphate dibasic; PBS), oxalic acid, sulfuric acid, phenol, and sodium pentobarbital, were purchased from Sigma Aldrich (St. Louis, MO, USA).

### 2.3. Obtention of the RS Sample

The evaluated RS ingredient was previously developed and reported by our group. Additional information about RS production and characteristics can be found in Suáred-Diéguez et al. [6]. Briefly, this RS material consisted of a type II RS product (retrograded starch) obtained under evaluated experimental optimal conditions previously reported by our group, as we mentioned before. The obtention procedure of the RS ingredient consisted of diverse steps. First, native starch isolation was conducted, followed by gelatinization and debranching processes with pullulanase. Finally, the retrogradation process (starch crystallization) was conducted.

Native starch isolation was conducted according to the method reported by Suárez-Diéguez et al. [15], based on the wet-grinding and leaching principle, by using nylon canvases. Beans were soaked overnight in a 0.1% sodium metabisulfite solution (1:3 *w*/*v*) at 20 °C. Afterward, the beans were wet grinded and an extraction process was conducted by applying the leaching principle through nylon canvases. The extracts were stored overnight at 8 °C, allowing them to generate a sediment, which was washed with sodium hydroxide (0.5%) until a clear material was reached. Finally, the isolated native starch was dried in a conventional oven at 40 °C for 30 h.

For the debranching process, the isolated native starch was previously gelatinized in a sodium acetate anhydrous buffer (0.1 mol/L; pH 5.0) at 95 °C for 15 min (10 g native starch/100 mL buffer). After that, the debranching process was performed by submitting the gelatinized native starch to a pullulanase treatment of the enzyme at 18 U/g of starch for 27 h at the optimal enzyme temperature (46 °C), reaching a 100% debranching capacity. Finally, the retrogradation step (starch crystallization process) was conducted at 20 °C for 6 days. The retrogradation process increased the RS content in the starch sample by approximately 42%, reaching a final RS content in the retrograded starch close to 65%. This RS ingredient obtained from creole faba beans showed a polymorphic crystallinity type B + V pattern (determined by X-ray diffraction), a crystallinity degree of 19.26% (determined by X-ray diffraction), an increased slow digestibility starch (SDS) fraction, and an in vitro digestibility reduction of approximately 50% (total digestibility of 49.6%). Additionally, the common native starch morphological structure disappeared in the RS material, being characterized by a more shriveled morphology generated by an amorphous, heterogeneous, irregular, and semicrystalline continuous network characteristic of retrograded starches (by SEM methodology). The whole detailed description of the obtention process, as well as the ingredient characteristics, can be found in our previous research on RS sample development, where creole RS obtention and characterization were performed [6].

### 2.4. Animal Acquisition and Husbandry

Wistar rats were used as experimental animal models. Healthy male animals (10–12 weeks of age) with a mean weight of 200–220 g were selected for this investigation (*n* = 30). The animals were acquired from Charles River Laboratories (Wilmington, MA, USA) and kept in the Bioterio del Instituto de Ciencias de la Salud of the Universidad Autónoma del Estado de Hidalgo (Bioterio of the Health Science Faculty of the Autonomous University of Estado de Hidalgo). Wistar rats were housed in polycarbonate cages (2 animals per cage), 6 mm thick (32 cm × 47 cm × 20 cm), and wood shavings were used as bedding. Animals were maintained under normal laboratory conditions of temperature (22 ± 2 °C), humidity, and a 12 h dark–light cycle. For feeding, access to water and food was provided ad libitum. A commercial standard rodent pelleted diet (chow 5001, LabDiet, Saint Louis, MO, USA) was provided for animal feeding. The diet pellets were composed of proteins (23.0%), lipids (4.5%), fiber (6.0%), minerals (8.0%), and carbohydrates (49%). Therefore, total calorie intake consisted of 28.5% protein, 13.5% lipid, and 58% carbohydrate, allowing an isocaloric diet across all groups (additional information in Table S1). Hence, the experimental intervention groups were fed the same isocaloric diet mixed with the RS sample based on retrograded starch from creole faba beans (15% or 30%; *w*/*w*), supplemented with casein, soy oils, and cellulose to respect the isocaloric macronutrients provided. To this end, the commercial diet chow 5001 was ground into a powder and mixed with the appropriate RS sample with low amounts of water (90% *w*/*v*) to generate a mixed mass (also mixed with the other supplements). Pellets were handcrafted and dried in an oven at 35 °C, reconstructing the pellets to be provided as food (as granulates). To determine the effect of the diets on the nutritional parameters and to monitor the experiment development, the weight of all the rats from all the experimental groups was monitored during the whole experiment.

### 2.5. Ethical Clearance

The experimental protocol was approved in 2018 by the Ethics Committee of the Bioterio del Instituto de Ciencias de la Salud of the Universidad Autónoma del Estado de Hidalgo (CIECUAL) (protocol code CIECUAL/018/2018; date of approval 18 October 2018). The animal management and all the experimental procedures applied were in accordance with the Official Mexican Normative NOM-062-ZOO-1999. Additionally, the study followed the guidelines for the care and management of laboratory experimental animals of the National Research Council’s Guide for the Care and Use of Laboratory Animals.

### 2.6. Establishment of Streptozotocin (STZ)-Induced Diabetes in Rats

Healthy male rats were given one week of acclimatization. After that, type 2 diabetes mellitus was induced according to Zhou et al. [16]. For this purpose, the diabetes condition was induced with intravenous administration of streptozotocin (STZ, 50 mg/kg of rat weight), and animals were maintained under overnight fasting. Additionally, intravenous citrate buffer (0.1 M) was administered to the non-diabetic rat groups (ND). To ascertain the existence of a diabetic condition (or non-diabetic condition), one week after diabetes induction (or controls), serum glucose levels in all experimental rats were quantified after overnight fasting conditions. Rats (*n* = 30) were randomly divided (simple randomization) into 5 groups, with 6 rats in each group (*n* = 6). Sample size was chosen according to the criteria reported by Kim et al. [17] and Zhou et al. [16]. Therefore, experimental groups were constituted according to physiological condition—non-diabetic (ND) or diabetic (D)—as well as whether they were treated with the ingredient (T) or non-treated (NT). Then, 5 different groups were constituted: Group I, non-diabetic rats without ingredient treatment (ND; healthy control group); Group II, non-diabetic rats with ingredient treatment consisting of 15% replacement (ND15); Group III, diabetic rats without ingredient treatment (DNT; control diabetic group); Group IV, diabetic rats with ingredient treatment consisting of 15% replacement (DT15; diabetic treated group); and Group V, diabetic rats with ingredient treatment consisting of 30% replacement (DT30; diabetic treated group). The experimental period, according to each group’s characteristics, lasted 30 days, comprising the whole intervention period. At the end experimental period, fasting glucose serum levels, cholesterol (CHO), triglycerides (TG), VLDL, LDL, HDL, glycosylated hemoglobin, Advanced Glycation End Products (AGEs), and liver and kidney antioxidant enzyme levels were measured.

### 2.7. Serum and Tissue Collection

After 30 days under experimental conditions (fed with or without RS material replacement), all rats were maintained under 12 h fasting conditions and anesthetized with sodium pentobarbital (1 g/L). Blood samples were collected through cardiac puncture and placed into tubes with anticoagulants to prevent sample hemolysis. Blood samples were centrifuged at 10,000 rpm for 15 min. Serum phase was collected and stored at −80 °C until further analyses. Finally, the rats were euthanized by cervical dislocation and immediately dissected to collect the liver and kidney organs. Remaining blood in the collected organs was removed by washing each organ individually with a physiological solution. The organs were stored in liquid nitrogen. For further analyses, the organs were chopped and homogenized with a phosphate buffer solution (50 mM, pH 7.4) and centrifuged at 10,000 rpm at 4 °C for 10 min; the supernatants were collected and stored at −80 °C.

### 2.8. Metabolic Biomarkers

The serum samples of all groups of animals collected after the fasting period (12 h) at the end of the experiment were used to analyze diverse metabolic biomarkers. Analyses were conducted on fasting glucose, cholesterol, triglycerides, HDL, LDL, and VLDL serum levels. An enzymatic-colorimetric commercial kit was used to measure the mentioned metabolic biomarkers, according to the manufacturer’s instructions (Wiener Laboratories S.A.I.C., Santa Fe, Argentina). Additionally, HDL and LDL analyses allowed to determine the LDL/HDL index. Glycosylated hemoglobin levels were measured using a Stanbio Glicohe-moglobina (Pre-Fil) kit (Stanbio Laboratory, Boerne, TX, USA).

Finally, the measurement of the AGEs was determined according to Ansari et al. [18], with a few modifications, based on the formation of 5-hydroxylmethyl-furfural (5-HMF) from glycated proteins. Briefly, after the previously mentioned blood sample centrifugation and plasma collection, the packed erythrocytes were collected and washed twice with normal saline solution and stored at −20 °C for 14 days. Just before the assay, the samples were allowed to defrost at room temperature (20 °C). An aliquot of 100 μL was mixed with 1.4 mL of distilled water and vortexed for 2 min. Then, 0.5 mL of an oxalic acid solution (0.5 M) was added, and the mixture was kept for 2 h in a boiling water bath. Immediately after, the tubes were cooled on ice for 10 min, and 1 mL of ice-cooled trichloroacetic acid (TCA, 40%) was added, mixed in a vortex for 2 min, and centrifuged at 2000 rpm for 1 min. The clear supernatant was collected, and 1 mL was treated with concentrated sulfuric acid (3 mL) and 80% phenol (0.05 mL) and allowed to stand for 30 min for color development. Finally, the absorbance of the generated 5-HMF was measured at 480 nm. The amount of HMF was calculated using the molar extinction coefficient value of 4 × 10^4^/cm × mol.

### 2.9. Nutritional Parameters

Diverse nutritional parameters were monitored along the experimental period to elucidate the effect of the diet intake with RS ingredient replacement, also considering the physiological condition (diabetic or non-diabetic). Therefore, commonly evaluated nutritional parameters—namely body mass gain (BMG), specific growth rate (SGR), metabolic growth rate (MGR) [19], and feed converting rate (FCR) [11]—were determined. For this purpose, the following equations were used:

BMG% = [final body mass (FBM) − initial body mass (IBM)/IBM] × 100(1)

SGR (% per day) = [(ln FBM in g) − (ln IBM in g)/number of trial days] × 100(2)

MGR (g kg0.8/day) = (BMG g)/{[(IBM g/1000)0.8 + (FBM g/1000)0.8]/2}/duration of the trial days(3)

FCR = feed intake/weight gain(4)

### 2.10. Glucose Homeostasis Analyses

#### 2.10.1. Postprandial Glucose Response

At the end of the experimental period (30 days), the animals were submitted to a 12 h fasting period before the postprandial response assay was conducted. Then, the corresponding diets were provided, and the animals were allowed to feed ad libitum for 30 min. After that, the food was removed, and the animals were not fed again. Blood samples (0.5 mL) were collected in tubes with heparin (as an anticoagulant) by capillary puncture of the saphenous vein in the tail of the animals every 30 min for 150 min. Subsequently, blood samples were centrifuged (5 min at 5000 rpm) and the serum phase was collected and frozen at 80 °C until further analysis. The glucose concentration for all the collected blood samples was evaluated to determine the postprandial glucose response using the commercial kit previously mentioned (Wiener Laboratories S.A.I.C. brand, Santa Fe, Argentina).

#### 2.10.2. Insulin Resistance Index

The triglyceride-glucose (TyG) index was used to evaluate insulin resistance [20]. This value results from the product of fasting serum concentrations of total triglycerides and glucose, expressed on a logarithmic scale. The following equation was used:

TyG = Ln [fasting triglycerides (mg dL) × fasting glucose (mg dL)/2](5)

#### 2.10.3. Oxidative Stress Biomarkers

The endogenous antioxidant enzyme activity of the obtained liver and kidney tissue homogenates from each experimental group was measured. Superoxide dismutase (SOD) was analyzed using the technique of Sun et al. [21]; catalase was performed following the method of Baudhuin et al. [22]. These measurements allowed cellular oxidative stress levels in diabetic animals (compared to non-diabetic animals) to be determined because of the chronic hyperglycemia.

### 2.11. Statistical Analyses

The statistical analyses of the results (obtained by triplication) were conducted using the statistical software Statgraphics Centurion XVI (Statistical Graphics Corp., Warrenton, VA, USA). One-way ANOVA, along with Duncan’s post-hoc tests (significance level of *p* ≤ 0.05), was used for mean comparisons.

## 3. Results and Discussion

### 3.1. Effect of the RS Ingredient on Nutritional Parameters

The food intake, weight gain, and body development in all the experimental groups were monitored during the whole experimental period to elucidate the effects of the RS ingredient intake on the animals’ nutritional parameters. These results are shown in Table 1. The non-diabetic animals (ND and ND15) showed weight gain and body development in concordance with the common animal physiology. These results indicate that the food composition and intake levels were appropriate for the animals’ growth, even with the RS material replacement (15%). However, it should be considered that these results are from male rats, so the response of females should be evaluated in further investigations to corroborate this trend regardless of animal sex.

By comparing the diverse experimental groups, differences can be found between the non-diabetic and diabetic groups. In this regard, the non-diabetic groups (ND and ND15) consumed significantly less food compared with the diabetic groups (DNT, DT15, DT30). However, no differences in weight gain were observed between the animal groups, except for DNT, which had significantly less weight gain compared with the other animal groups, regardless of the applied treatment or physiological condition. The animals gained close to 11% of their initial weight (10.53–12.36%), whereas the DNT group gained only 3.8% of their initial weight; furthermore, two of the animals experienced weight loss. The same trend was found for the other nutritional parameters (BMG, SGR and MGR), with no significant differences between groups, except for the lower values in DNT.

The glucose intake of the DNT group was not properly managed, and therefore the animals’ development was affected, possibly leading to muscular mass degradation [23]. Conversely, the diabetic animals treated with the RS ingredient did not show this trend, despite the applied treatment. These results suggest a potential functional effect of the RS sample. The RS ingredient resulted in an improvement in the metabolic harnessing of glucose in diabetic animals, consequently allowing for normal growth (similar to non-diabetic animals). These results were also in concordance with the improved postprandial glycemia determined for DT15 and DT30 compared with the DNT group (Section 3.4). On the other hand, although clear differences were observed for FCR (Feed Conversion Rate), no significant differences were found between all the experimental groups, probably due to the DNT group (Table 1). Nevertheless, noticeably higher FCR values were calculated for DT15 and DT30 compared with DNT or DT15, suggesting more efficient food conversion in the non-diabetic animals. These results are in concordance with the physiological conditions of the animals, where higher metabolic efficiency was observed in the non-diabetic groups, regardless of whether the ingredient was consumed. In this context, a similar trend was observed in healthy and diabetic animals fed RS from Negro Jamapa beans [11]. The insulin impediment under diabetic conditions leads to frequent hyperglycemia, unbalancing glucose homeostasis [24]. Therefore, worsening energy exploitation may negatively affect the individual’s growth [11]. This pathological condition activates the conversion of amino acids into glucose through the hepatic gluconeogenesis pathway, avoiding the bioavailability of these compounds and concluding in possible muscle degradation [23].

Conversely, attenuated glucose release during gastrointestinal digestion triggers a reduced glycemic response, as has been observed with legume RS ingredients [11]. Human clinical trials also show a reduced postprandial glycemic response associated with RS intake, a consequence of the digestion impairment of the RS [25,26,27]. Consequently, glucose management can last up 6 h (5–7 h) [26]. Therefore, on the one hand, an improved postprandial glycemic response leads to lower insulin secretion and inhibition of hepatic gluconeogenesis, and therefore, a progressive energetic management of glucose intake [28]. In this context, the improved nutritional parameters of DT15 and DT30 compared with DNT are explained by attenuated hyperglycemic responses, which allow for improved glucose and energy management and better prognostics in the nutritional parameters. Additionally, RS with SDS fractions allows for attenuated and extended glucose release compared with starch. Therefore, an additional but lasting glucose supply enables glucose management beyond the early postprandial glycemic response (0–3 h), hence, increasing the amount of metabolic used glucose under diabetic conditions [25], which also explains the improved nutritional prognostics in the diabetic treated group compared with the non-treated diabetic group. In this context, the common physiological response in diabetic conditions of adipose lipolysis activation and free fatty acids postprandial rebounds can be avoided by lasting glucose management [27], as can be found for RS intake in human trials [25]. Therefore, in summary, lasting glucose management derived from RS intake may attenuate various common physiological responses associated with the diabetic condition, such as gluconeogenesis activation due to inefficient glucose cellular intake, increased insulin resistance due to lower insulin receptor sensibility, or free fatty acids rebound from adipose triglycerides hydrolysis caused by impaired glucose cellular intake [25,26,27]. Obviously, all these anabolic metabolic responses are associated with energy expenditure in diabetic conditions that can compromise nutritional parameters, such as muscular degradation or lipid accumulation [23,29]. Hence, based on the results obtained in this investigation, it is suggested that consumption of the RS ingredient obtained from creole faba bean may influence the energy and glucose homeostasis in diabetic individuals, as has been previously observed for other RS sources [11,30]. However, additional analyses, such as of lipid accumulation levels in adipose tissue, free fatty acid plasma levels, and genetic expression of diverse biomarkers associated with glucose metabolism, should be conducted in future studies to confirm these results.

### 3.2. Effect of the RS Ingredient on Glucose Metabolism Biomarkers

To determine the diet effect on glucose homeostasis, the serum glucose levels under fasting conditions were measured at the beginning and end of the experimental period (Table 2). Then, one week after diabetic induction at the beginning of the experimental period, lab rats were submitted to a 12 h fasting period and serum glucose levels were quantified to ensure the diabetic condition was reached. The induced diabetic groups showed hyperglycemia (higher than 11.1 mmol/L) with no significant differences among them (23.73 ± 3.62, 21.94 ± 2.00, and 24.76 ± 2.94 mmol/L for DNT, DT15, and DT30, respectively). Conversely, the non-diabetic groups showed normal glycemia (4.71 ± 0.89 and 4.86 ± 1.16 mmol glucose/L for ND and ND15, respectively). These results confirm the induction of diabetes within DNT, DT15, and DT30 and a healthy condition within ND and ND15.

The fasting glucose serum levels (12 h fasting) at the end of the experimental period resulted in noticeably higher levels for DNT, DT15, and DT30 (27.81 ± 3.09 mmol/L, 16.89 ± 2.29 mmol/L, and 19.36 ± 2.81 mmol/L, respectively) compared with the non-diabetic groups, remaining over normal physiological values. However, the diabetic-treated groups DT15 and DT30 showed significantly lower fasting glucose levels compared to DNT, suggesting the potential effect of the RS ingredient. In this context, fasting glucose at the end of the experimental period for DT15 and DT30 resulted in a significant reduction of approximately 22% compared with the initial glucose levels (21.94 ± 2.00 vs. 16.89 ± 2.29 mmol/L, and 24.76 ± 2.94 vs. 19.36 ± 2.81 mmol/L, respectively), whereas no differences were found in the DNT group (23.73 ± 3.62 vs. 27.81 ± 3.09 mmol/L). Fasting glucose improvement derived from RS intake in diabetic lab rats has been previously reported [11,16,31], being in concordance with the present investigation. Also, attenuated and reduced postprandial glycemia response are the most common effects of RS consumption in human trials [25].

In line with fasting glucose levels at the end of the experimental period, glycosylated hemoglobin % levels were significantly lower in DT15 and DT30 than in DNT. In this context, glycosylated hemoglobin levels were reduced by approximately 30% in DT15 and DT30 compared with the diabetic control group. A similar trend was also observed for the AGEs levels. Nevertheless, the diabetic groups resulted in noticeably higher values of glycosylated hemoglobin % and AGEs compared with the non-diabetic group. Therefore, the RS ingredient intake improved glucose metabolism, although it did not allow them to reach normal conditions.

The consumption of RS modulates diabetic biomarkers significantly reduced the fasting glucose and the glycosylated hemoglobin levels, as has been demonstrated in a meta-analysis of human clinical trials of diabetic individuals [32]. On the other hand, although AGE levels of diabetic individuals treated with RS have been less investigated, the consumption of this ingredient may be associated with lower AGEs values under this pathological condition [33]. During the approximately 3 months that erythrocytes circulate in the plasma, progressive binding with blood glucose molecules occur [34]. Therefore, higher glycemia is associated with increased levels of glycosylated hemoglobin [35]. Additionally, AGEs are also a consequence of chemical bonds between blood molecules (proteins, lipids, and nucleic acids) and blood glucose. Hence, hyperglycemic condition triggers incremented glucose expositions and AGE formations [36]. The attenuated postprandial glycemic response and improved glucose management in the diabetic treated groups, as can be observed in fasting glycemia, resulted in a general lower glycemia (during both postprandial and fasting conditions), associated with the reduced glycosylated hemoglobin and AGE levels observed [35,36]. Besides, AGEs increment in the diabetic groups compared with non-diabetic groups were also in concordance with the antioxidant liver enzymes results (Section 3.6), since ROS increments are associated with AGE augmentations [37].

These results regarding glucose metabolic biomarkers suggest a positive impact on glucose homeostasis derived from the investigated ingredient intake, mainly regarding the fasting serum glucose levels, being in concordance with the postprandial glucose kinetics (Section 3.4). Therefore, the mechanism of action of the RS ingredient could be explained not only by a reduced and attenuated digestibility and high RS content but also by a direct impact in the glucose metabolism pathway and homeostasis, as has been previously suggested for other RS ingredients [6,16]. Nevertheless, although glucose metabolism is enhanced by RS intake, the diabetic condition is not reversed, allowing improved metabolic biomarkers but not reaching normal parameter values, as could be observed for the non-diabetic animals.

### 3.3. Effect of the RS Ingredient on Lipid Metabolism Biomarkers

The total cholesterol, triglycerides, HDL, and VLDL serum level was slightly increased in the diabetic animal groups compared with non-diabetic rats (Table 3). However, LDL levels were not significantly different regardless of the experimental group, and as a consequence similar results were determined for the LDL/HDL index. Additionally, similar values were observed among diabetic animals, with a non-significant trend toward an increase in these biochemical biomarkers in the non-treated diabetic group. Conversely, previous studies reported improvements in lipid metabolism derived from RS intake in diabetic lab rats [11,16,31]. Therefore, the results of this study suggest that the incorporation of RS material into the animal diet may not impact the lipid profile, despite modulating glucose metabolism. Nevertheless, it must be considered that, derived from insulin resistance, a TG and VLDL synthesis promotion occurs in the liver, as well as CHO increments [38,39], and these may explain the trend of slight increments of TG, VLDL, and CHO serum levels in diabetic individuals compared with non-diabetics. In this regard, it must be considered that treated diabetic animals in this study did not get a healthy TyG Index (Section 3.5), resulting in an attenuated but existing insulin resistance, which can trigger increments in TG, VLDL, and CHO, as we commented above.

In addition, it must be considered that diabetic animals consume approximately twice the amount of food as non-diabetic individuals, triggering higher total fat consumption. In this context, it is noticeable that the TyG index, despite being at healthy values for the non-diabetic animals, resulted in high values, a consequence of the high serum triglycerides (also in the treated non-diabetic group), suggesting that the provided diet was high in calories, probably allowing for the lipogenesis processes in all the animal groups. In this regard, the observed results for the diabetic treated animals could be a consequence of food over-intake by these groups, which could be distorting the possible effect of the RS ingredient on lipid metabolism, since higher lipid and calorie intake are observed, together with a weak effect of the RS intake on lipid metabolism.

### 3.4. Effect of the RS Ingredient on Glucose Homeostasis: Postprandial Response

**Figure 1:**
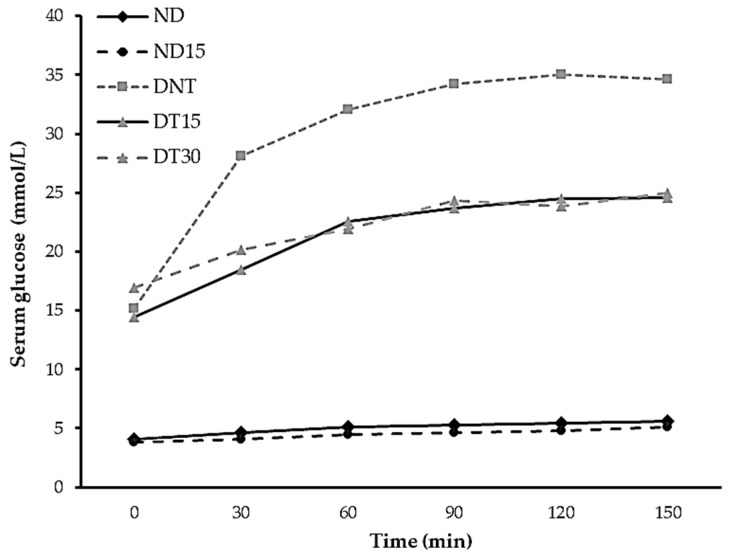
Postprandial glucose serum levels of different experimental groups fed or non-fed with the RS ingredient replacement. ND, non-diabetic without treatment group; ND15, non-diabetic treated with 15% of RS group; DNT, diabetic non-treated group; DT15, diabetic treated with 15% of RS group; DT30, diabetic treated with 30% of RS group.

Once the end of the experimental period was reached (30 days), the animals were submitted to a 12 h fasting period. Afterward, the animals were fed ad libitum. Then, fasting was re-introduced and postprandial glycemic response was monitored for 150 min (Figure 1). The non-diabetic animals showed a typical physiological response, without differences between the ND and ND15 groups. Conversely, DNT, DT15, and DT30 showed an altered glucose postprandial response, with a glucose peak at 120 min in all cases, indicating a typical diabetic response. However, noticeable differences were observed between the treated and non-treated diabetic groups. Treated diabetic animals tended to have lower serum glucose levels (DT15 and DT30) without differences among them, but they showed significant difference with DNT (24.56 ± 7.50 and 25.02 ± 3.54 vs. 34.65 ± 1.89 mmol/L, respectively). After the experimental period, the DT15 group showed a reduction in the hyperglycemic condition of 29% and DT30 showed 28% compared to the DNT group. These results demonstrate a reduced and attenuated postprandial response due to RS intake, which is in concordance with the lower in vitro digestibility previously observed for the studied sample [6]. Similar trends have been observed in the postprandial glucose response in diabetes-induced lab rats treated with RS [11,16,31] and in human clinical trials [40].

Postprandial glucose response is dependent on the glucose release kinetics of the intake food [28]. Nevertheless, the reduction in blood glucose levels in rats fed with RS may also be a consequence of the modulation of the expression of genes related to glucose metabolism, as well as improvements in pancreatic function [31]. On the other hand, RS intake by diabetic animal models showed down-regulated expression levels of the key regulatory enzymes of the gluconeogenesis pathway, such as glucose-6-phosphatase (G6PC1) and phosphoenolpyruvate carboxykinase (PEPCK) [16]. The attenuated postprandial glycemic response of the diabetic-treated groups of this study (DT15 and DT30) is a consequence of the reduced and progressive glucose release during gastrointestinal digestion, also confirmed by in vitro digestion assays, due to the high content of RS in the functional starch sample studied [6]. However, the improvement in the postprandial glucose responses of the DT15 and DT30 groups cannot be uniquely explained by an attenuated postprandial glycemic response since the total digestibility of the RS ingredient was reduced by approximately 50%; additionally, no differences were determined between DT15 and DT30, indicating that the reduced digestibility of the RS ingredient is not the only factor of its functionality. Hence, a regulatory effect on glucose metabolism mediated by the consumption of the RS ingredient is suggested, which enhances glucose metabolic homeostasis, as previously observed by other authors in various RS samples. Therefore, further experiments focusing on gene expression related to hepatic gluconeogenesis and glucose metabolism pathways are needed to elucidate the contribution of the evaluated RS ingredient to glucose homeostasis. Additionally, muscle glucose intake (GLUT-4 genetic expression, muscular glycogen storage, and related enzyme genetic expression), as well as a potential prebiotic effect, should also be investigated to achieve a comprehensive understanding of the RS functional mechanisms beyond reduced starch digestibility.

### 3.5. Effect of the RS Ingredient on Glucose Homeostasis: Insulin Resistance Index

TyG index (the triglycerides and glucose index) has been proposed as a biomarker for predictable insulin resistance, where 8.7–8.8 indicates prediabetic and diabetic conditions [20]. The results of the TyG index followed the previously observed trend for fasting glucose levels. Hence, insulin indices of 8.68 ± 0.26 and 8.54 ± 0.23 (ND and ND15, respectively) were determined in the non-diabetic groups after the experimental period. On the contrary, the diabetic groups displayed TyG values above 8.8 (Table 2), indicating potential insulin resistance. However, slightly reduced index values and therefore a significantly better prognosis were observed for the DT15 and DT30 groups (9.88 ± 0.60 and 9.94 ± 0.30, respectively) compared to the DNT group (10.49 ± 0.11). The results suggest that improvements in insulin resistance in diabetic conditions can be achieved with RS intake, principally by enhancing glucose fasting levels but not allowing them to get to normal healthy levels. In this context, reduced insulin resistance enhances cell glucose intake, which is in concordance not only with the improved nutritional parameters observed for the diabetic treated groups but also with the reduced biomarkers associated with glucose metabolism (glycosylated hemoglobin and AGEs) [36].

### 3.6. Effect of the RS Ingredient on Oxidative Stress

Oxidative stress was evaluated throughout by quantifying superoxide dismutase (SOD) and catalase (CAT) in the liver and kidneys as metabolic stress biomarkers. In this context, mixed results were observed (Table 4). No statistical differences were found between the different groups in the liver. However, an upward trend was observed in diabetic groups, with the highest values recorded in animals of DNT group. Conversely, SOD activity in the kidneys was statistically lower in the non-diabetic groups compared with the DNT group, but no statistical differences were observed for the diabetic-treated groups (DT15 and DT30), ND, ND15, and DNT. On the other hand, CAT in the liver was significantly lower in non-diabetic groups compared to diabetic groups (without differences between DNT, DT15, and DT30); meanwhile, CAT in the kidneys of all the experimental groups was significantly different from DNT, although no differences were observed among ND, ND15, DT15, and DT30.

Diabetic condition is frequently associated with a decrease in cellular endogenous antioxidant response. In this context, a maintained hyperglycemic condition is related to higher radical oxygen species (ROS) [37]. As a consequence, the harmful effects of free radicals can be observed. Hence, chronic hyperglycemia can inactivate antioxidant enzymes, such as SOD or CAT, due to structural modifications and also reduce their genetic expression [41,42]. Nevertheless, it must be considered that controversial results can be found in the scientific literature, and some diabetic experimental models show no differences between the diabetic and the non-diabetic groups [43,44]. In this context, short-term oxidative status can trigger an increase in antioxidant enzyme expression, such as CAT, as a compensatory mechanism to increased ROS exposure levels [45]. In this regard, although previous research by our group showed that induced diabetic Wistars rats treated with RS from the Negro Jamapa bean displayed decreased liver CAT activity, probably due to increments in ROS levels and confirmed by higher malonaldehyde levels, the diabetic-treated group showed slight enhancements compared to the diabetic non-treated group [11], and this trend was not observed in this study. Higher liver CAT activity was observed in diabetic conditions, despite the applied treatment. The results of this study suggest that liver CAT overexpression in diabetic groups was a consequence of a compensatory mechanism driven by increased oxidative stress status derived from the noticeable hyperglycemic conditions, although maybe the experiment extension was not enough to observe a clear CAT degradation or genetic expression inhibition process [42,45]. Additionally, it also explains the elevated activity of kidney CAT in the diabetic non-treated group, a consequence of the compensatory mechanism. In this context, Lucchesi et al. monitored CAT and SOD expression, among other enzymes, in diabetic lab rats and observed a time-dependent expression, characterized by an increasing period (1–3 months) followed by a decreasing period (3–6 months) for CAT [46]. Additionally, Kakkar et al. [47] observed increments in CAT activity after the first week of induced diabetic condition in lab rats compared to the controls (non-diabetic), observing CAT decreases in week 5, agreeing with this study.

In this study, whereas CAT was augmented in diabetic conditions, liver SOD activity was not significantly different among all the experimental groups (diabetic and non-diabetic), although greater values were observed in the diabetic non-treated group, as well as in the kidney (where the diabetic condition tended to increase SOD activity). Kakkar et al. [47] reported that after 1 week of diabetes induction, lab rats showed increased values of SOD compared with non-diabetic individuals, which is in concordance with our study. Conversely, Lucchesi et al. observed maintained lower levels of liver SOD activity in diabetes [46].

Previous studies showed that RS consumption from beans improved the antioxidant status of the liver and kidneys [11]. The RS ingredient intake evaluated in this study may contribute to enhanced antioxidant status, although further analyses should be conducted with an increased experimental period and include other antioxidant enzymes, such as glutathione peroxidase (GPx), as well as measurements of lipid peroxidation (malondialdehyde levels).

## 4. Conclusions

RS material obtained from creole faba bean was analyzed for the first time in preclinical studies as a potential functional ingredient that could enhance the hyperglycemic postprandial response in diabetic conditions. Because of the lower and attenuated glycemic response, as well as improvements in diabetic biomarkers (reduced glycosylated hemoglobin % and AGEs), enhancements in the nutritional parameters and a better prognosis in resistance insulin index were observed. Additionally, due to the improved nutritional parameters in the diabetic treated group, the results suggest enhancements in glucose management and metabolism homeostasis beyond just an attenuated postprandial response. However, although the nutritional parameters clearly show an effect of RS consumption in diabetic condition management, limitations must be considered, since no genetic expression was evaluated. In addition, only males participated in this study; it is necessary to conduct further research with females to confirm the effect of RS in a non-gender-dependent way. Therefore, further studies must be conducted to completely elucidate the impact of RS ingredient consumption on glucose metabolic homeostasis, such as its effect on the hepatic gluconeogenesis regulation through gene expression levels, circulating blood insulin levels, or muscle glucose intake through intramuscular glycogen levels or GLUT-4 transport expression. Also, further investigations should be carried out to evaluate its full functional potential, including its potential prebiotic effect.

Therefore, preclinical results suggest that creole faba beans are a suitable source for producing potential functional RS ingredients, and the evaluated RS sample is a suitable potential functional ingredient for further clinical evaluation related to diabetic condition management.

## Figures and Tables

**Table 1:** The effect of RS derived from creole faba beans on nutritional parameters; BMG, SGR, MGR, and FCR; in non-diabetic and diabetic rats after 30 days of dietary intervention (mean value ± S.D.).

Group	Initial Weight (g)	Final Weight (g)	Weight Gain (g)	BMG (%)	SGR (%/Day)	MGR (g/Kg Day)	Feed Intake (g)	FCR
ND	321.00 ± 27.33	360.67 ± 32.93 ^a^	39.67 ± 8.73 ^a^	12.33 ± 2.32 ^a^	0.39 ± 0.07 ^a^	3.12 ± 0.57 ^a^	166.80 ± 35.39 ^b^	4.43 ± 1.60 ^a^
ND15	312.00 ± 34.39	346.67 ± 27.25 ^a^	34.67 ± 20.35 ^a^	11.59 ± 7.86 ^a^	0.36 ± 0.23 ^a^	2.86 ± 1.77 ^a^	143.78 ± 39.51 ^b^	4.69 ± 1.35 ^a^
DNT	236.43 ± 18.90	245.43 ± 8.85 ^b^	9.00 ± 19.84 ^b^	4.33 ± 9.18 ^b^	0.13 ± 0.30 ^b^	0.98 ± 2.26 ^b^	249.44 ± 13.45 ^a^	−1.26 ± 30.73 ^a^
DT15	232.29 ± 29.83	260.57 ± 33.92 ^a,^*	28.29 ± 12.54 ^a,^*	12.27 ± 5.38 ^a,^*	0.38 ± 0.16 ^a,^*	2.88 ± 1.20 ^a,^*	269.67 ± 20.33 ^a^	11.45 ± 5.37 ^a^
DT30	243.27 ± 23.03	269.33 ± 26.45 ^a,^*	25.67 ± 8.29 ^a,^*	10.00 ± 2.77 ^a,^*	0.32 ± 0.11 ^a,^*	2.40 ± 0.87 ^a,^*	258.64 ± 12.46 ^a^	12.21 ± 4.90 ^a^

FCR = food conversion ratio; BMG = body mass gain; SGR = specific growth rate; MGR = metabolic growth rate. ND, non-diabetic without treatment group; ND15, non-diabetic treated with 15% of RS group; DNT, diabetic non-treated group; DT15, diabetic treated with 15% of RS group; DT30, diabetic treated with 30% of RS group. ^a,b^ indicate significant differences between samples in the same column by Duncan’s post-hoc test (*p* ≤ 0.05). * Indicate significant differences between diabetic non-treated (diabetic control) and diabetic treated group values by Duncan’s post-hoc test (*p* ≤ 0.05).

**Table 2:** Effect of RS from creole faba beans consumption on glucose metabolism parameter biomarkers (initial glucose, final glucose, glycosylated hemoglobin, AGEs levels), as well as the TyG index, in non-diabetic and diabetic rats after 30 days of dietary intervention (mean value ± S.D.).

Group	Initial Glucose (mmol/L)	Final Glucose (mmol/L)	Glycosylated Hemoglobin (%)	AGEs µg 5-HMF/mg Protein	TyG Index
ND	4.71 ± 0.89 ^b^	5.34 ± 0.55 ^d^	5.34 ± 0.59 ^c^	0.21 ± 0.05 ^b^	8.68 ± 0.26 ^c^
ND15	4.86 ± 1.16 ^b^	5.21 ± 0.25 ^d^	5.02 ± 0.70 ^c^	0.22 ± 0.05 ^b^	8.54 ± 0.23 ^c^
DNT	23.73 ± 3.62 ^a^	27.81 ± 3.09 ^a^	13.78 ± 2.29 ^a^	0.45 ± 0.08 ^a^	10.49 ± 0.11 ^a^
DT15	21.94 ± 2.00 ^a^	16.89 ± 2.29 ^c,^*	9.73 ± 0.82 ^b^	0.36 ± 0.09 ^a^	9.88 ± 0.60 ^b^
DT30	24.76 ± 2.94 ^a^	19.36 ± 2.81 ^b,^*	9.01 ± 2.47 ^b^	0.35 ± 0.08 ^a^	9.94 ± 0.30 ^b^

AGEs = Advanced Glycation End Products; 5-HMF = 5-Hydroxymethilfurfural. ND, non-diabetic without treatment group; ND15, non-diabetic treated with 15% of RS group; DNT, diabetic non-treated group; DT15, diabetic treated with 15% of RS group; DT30, diabetic treated with 30% of RS group. ^a,b,c,d^ indicate significant differences between samples in the same column by Tukey post-hoc test (*p* ≤ 0.05). * indicate significant differences between initial and final glucose values by Tukey post-hoc test (*p* ≤ 0.05).

**Table 3:** The effect of RS derived from creole faba beans on lipid profile biomarkers and LDL/HDL index in non-diabetic and diabetic rats after 30 days of dietary intervention (mean value ± S.D.).

Group	CHO (mmol/L Blood)	TG (mmol/L Blood)	HDL (mmol/L Blood)	VLDL (mmol/L Blood)	LDL (mmol/L Blood)	LDL/HDL Index
ND	2.86 ± 0.28 ^b^	1.14 ± 0.13 ^b^	0.96 ± 0.15 ^c^	0.23 ± 0.03 ^b^	1.67 ± 0.43 ^a^	1.82 ± 0.70 ^a^
ND15	2.95 ± 0.30 ^b^	1.16 ± 0.05 ^b^	1.04 ± 0.08 ^b,c^	0.23 ± 0.01 ^b^	1.67 ± 0.28 ^a^	1.61 ± 0.30 ^a^
DNT	3.71 ± 0.42 ^a^	1.58 ± 0.25 ^a^	1.39 ± 0.12 ^a^	0.32 ± 0.05 ^a^	2.00 ± 0.31 ^a^	1.45 ± 0.19 ^a^
DT15	3.41 ± 0.23 ^a^	1.46 ± 0.15 ^a^	1.27 ± 0.25 ^b^	0.29 ± 0.03 ^a^	1.85 ± 0.43 ^a^	1.56 ± 0.66 ^a^
DT30	3.72 ± 0.18 ^a^	1.50 ± 0.19 ^a^	1.38 ± 0.24 ^a,b^	0.30 ± 0.04 ^a^	2.03 ± 0.29 ^a^	1.54 ± 0.56 ^a^

CHO = total cholesterol; TG = total triglycerides; HDL = total high-density lipoproteins; VLDL = total very low-density lipoproteins; LDL = total low-density lipoproteins. ND, non-diabetic without treatment group; ND15, non-diabetic treated with 15% of RS group; DNT, diabetic non-treated group; DT15, diabetic treated with 15% of RS group; DT30, diabetic treated with 30% of RS group. ^a,b,c^ indicate significant differences between samples in the same column by Duncan’s post-hoc test (*p* ≤ 0.05).

**Table 4:** Effect of RS from creole faba beans consumption on glucose metabolism oxidative stress biomarkers in non-diabetic and diabetic rats after 30 days of dietary intervention (mean value ± S.D.).

Groups	Liver		Kidney	
	SOD (U/mg Protein)	CAT (K/s/mg Protein)	SOD (U/mg Protein)	CAT (K/s/mg Protein)
ND	4.92 ± 0.58 ^a^	9.77 ± 2.55 ^b^	7.83 ± 3.44 ^a,b^	5.08 ± 1.60 ^b^
ND15	5.11 ± 2.80 ^a^	10.29 ± 0.78 ^b^	6.48 ± 2.53 ^b^	5.43 ± 1.06 ^b^
DNT	6.72 ± 0.93 ^a^	16.87 ± 2.40 ^a^	11.39 ± 4.02 ^a^	9.18 ± 3.42 ^a^
DT15	4.99 ± 1.06 ^a^	14.01 ± 3.39 ^a^	9.93 ± 3.30 ^a,b^	5.56 ± 1.92 ^b^
DT30	5.69 ± 3.32 ^a^	17.19 ± 3.95 ^a^	9.87 ± 1.26 ^a,b^	5.50 ± 2.28 ^b^

SOD = superoxide dismutase; CAT = catalase. ND, non-diabetic without treatment group; ND15, non-diabetic treated with 15% of RS group; DNT, diabetic non-treated group; DT15, diabetic treated with 15% of RS group; DT30, diabetic treated with 30% of RS group. ^a,b^ indicate significant differences between samples in the same column by Tukey post-hoc test (*p* ≤ 0.05).

## Data Availability

The data presented in this study are available on request from the corresponding authors. Data is unavailable due to privacy restrictions.

## Supplementary Materials

The following supporting information can be downloaded at https://www.mdpi.com/article/10.3390/nu17233807/s1, Table S1: Composition of the diet provided to each animal experimental group (g compound/kg diet) during the whole experiment period.

## Abbreviations

The following abbreviations are used in this manuscript:

AGEs	Advanced Glycation End Products
BMG	Body mass gain
CAT	Catalase
CHO	Cholesterol
DNT	Diabetic non-treated (control) group
DT15	Diabetic treated 15% RS replacement group
DT30	Diabetic treated 30% RS replacement group
FCR	Food conversion ratio
HDL	High-density lipoproteins
LDL	Low-density lipoprotein
MGR	Metabolic growth rate
ND	Non-diabetic control group
ND15	Non-diabetic treated 15% RS replacement group
RS	Resistant starch
SGR	Specific growth rate
SOD	Superoxide dismutase
TG	Triglycerides
VLDL	Very low-density lipoproteins
