# Supplementary material for: A New Resistant Starch Material Obtained from Faba Beans (Vicia faba L. Creole): Potential Modulation of the Diabetic Condition in Diabetic Wistar Rat Model
Source: Nutrients. 2025 Dec 4;17(23):3807. doi: 10.3390/nu17233807 (PMC12693899; doi:10.3390/nu17233807)
Supplement: Supplementary file 1 [file nutrients-17-03807-s001.zip › nutrients-4004175-supplementary.pdf]

Table S1. Composition of the diet provided to each animal experimental group (g compound/kg diet) during the whole experiment period.

	Experimental groups				
	**ND**	**ND15**	**DNT**	**DT15**	**DT30**
Chow Diet 5001	1000	750	1000	750	513
Casein	0	82	0	82	153
Soybean oil	0	20	0	20	34
Fiber (cellulose)	0	15	0	15	29
Mineral mixture[a]	35	35	35	35	35
Vitamin mixture[b]	10	10	10	10	10
Choline chloride	2.5	2.5	2.5	2.5	2.5
L-cysteine	3	3	3	3	3
RS Ingredient	0	150	0	150	300

ND, non-diabetic without treatment group; ND15, non-diabetic treated with 15% of RS group; DNT, diabetic non treated group; DT15, diabetic treated with 15% of RS group; DT30, diabetic treated with 30% of RS group.

[a] AIN-93 mineral mixture

[b] AIN-93 vitamin mixture
